# Glycogen metabolism has a key role in the cancer microenvironment and provides new targets for cancer therapy

**DOI:** 10.1007/s00109-015-1377-9

**Published:** 2016-02-17

**Authors:** Christos E. Zois, Adrian L. Harris

**Affiliations:** Molecular Oncology Laboratories, Department of Oncology, Weatherall Institute of Molecular Medicine, John Radcliffe Hospital, Oxford University, Oxford, OX3 9DS UK

**Keywords:** Glycogen metabolism, Cancer therapy, Tumour Microenvironment, Hypoxia, Radiation

## Abstract

Metabolic reprogramming is a hallmark of cancer cells and contributes to their adaption within the tumour microenvironment and resistance to anticancer therapies. Recently, glycogen metabolism has become a recognised feature of cancer cells since it is upregulated in many tumour types, suggesting that it is an important aspect of cancer cell pathophysiology. Here, we provide an overview of glycogen metabolism and its regulation, with a focus on its role in metabolic reprogramming of cancer cells under stress conditions such as hypoxia, glucose deprivation and anticancer treatment. The various methods to detect glycogen in tumours in vivo as well as pharmacological modulators of glycogen metabolism are also reviewed. Finally, we discuss the therapeutic value of targeting glycogen metabolism as a strategy for combinational approaches in cancer treatment.

## Glycogen metabolism

Glycogen is the storage form of glucose in cells and is essential for energy supply and glucose homeostasis. The discovery of glycogen in liver in 1857 is attributed to Claude Bernard [1]. The general mechanism of glycogen synthesis and degradation is the same in all tissues, whilst the regulation of glycogen metabolism differs.

**Fig. 1:**
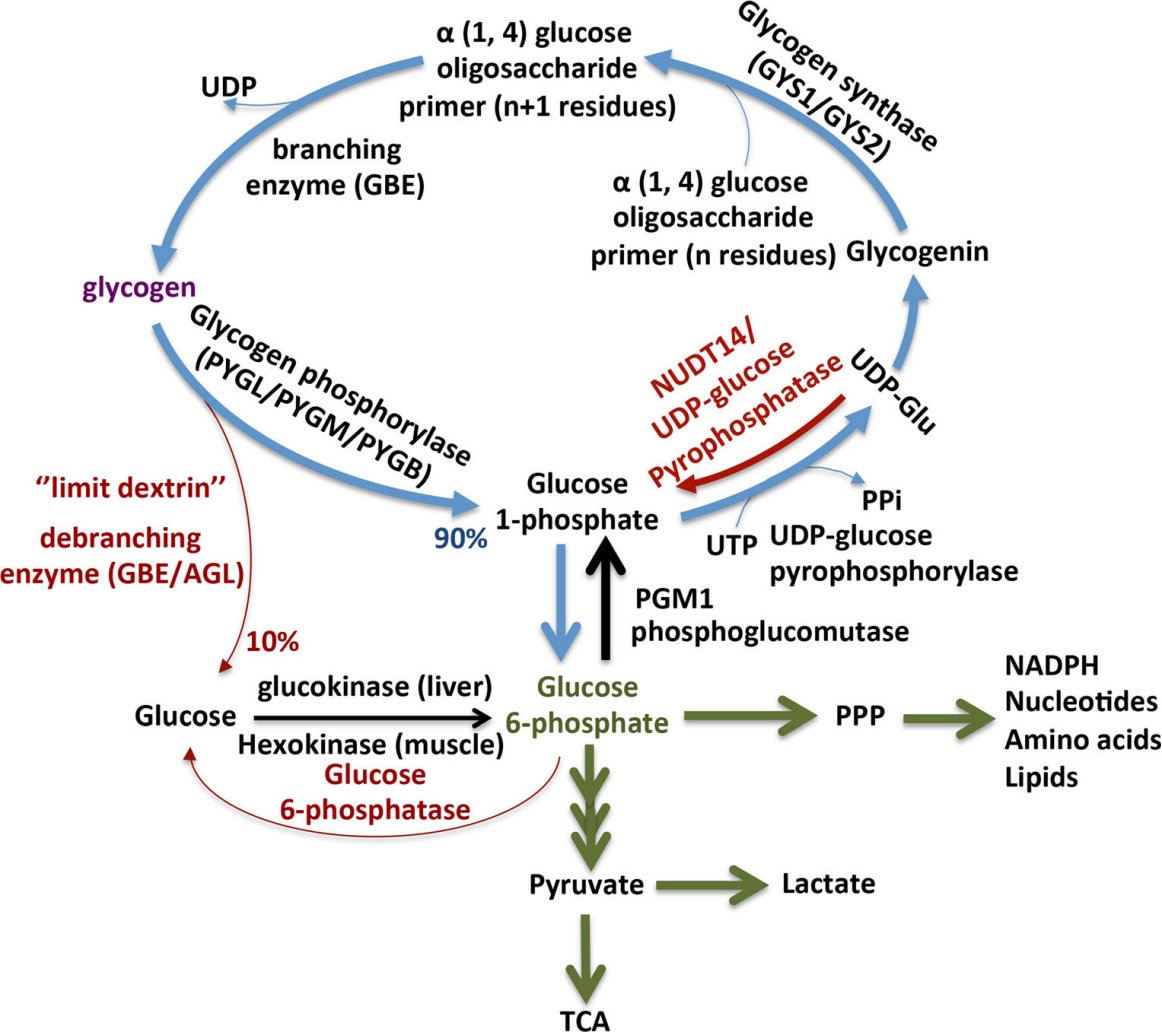
Key pathways of glycogen synthesis and metabolism. Glycogen synthesis requires formation of UDP-glucose from glucose-1-phosphate and UTP to be catalysed UDP-glucose pyrophosphorylase. Glycogenin initiates the first step of glycogen synthesis by self glycosylation of a short 8–12 glucose oligosaccharide primer. Glycogen synthase (GYS) elongates the glucose oligossacharide primer, which utilises UDP-glucose as the glucosyl donor. Glycogen branching enzyme (GBE) catalyses the transfer of α-1,4-linked glucose units from the outer ‘non-reducing’ end of a growing glycogen chain into an α-1,6 position of the same or neighbouring chain. Glycogen degradation requires the synchronous activation of the glycogen phosphorylase and the bifunctional enzyme glycogen debranching enzyme. Glycogen phosphorylase releases glucose-1-P from the terminal alpha-1,4-glycosidic bond, and the glycogen debranching enzyme catalyses the removal of the branches. DBE transfers three glucose blocks to another glycogen chain, and then hydrolytically cleaves the remaining glucose of the branch, generating free glucose. Glycogen-derived glucose-1-P and free glucose can enter the glycolytic or the pentose phosphate pathway (PPP)

Glygogen synthesis is performed in the cytosol from extracellular glucose transported into the cells through glucose transporters or from an indirect pathway where lactate and amino acids can be used. Figure 1 provides a general schematic presentation of the glycogen pathway. The first step of glycogen synthesis consists of autoglucosylation of the core protein, glycogenin, which provides an oligosaccharide primer. To this oligosaccharide primer, glycogen synthase elongates the glucose chain by attaching uridine diphosphate (UDP)-glucose units through α-1,4 glycocidic linkage. Next, when the elongating chain reaches around 12 glucose units, then a glycogen branching enzyme transfers a chain of seven units to an adjacent chain via α-1,6 glycosidic bond. Glycogen synthase elongates the glycogen chain, whilst the glycogen branching enzyme produces new branches.

Glycogen breakdown requires synchronous activities of glycogen phosphorylase and the bifunctional glycogen debranching enzyme. Glycogen phosphorylase, which is the rate-limiting enzyme of glycogenolysis, cleaves the α-1,4 linkage to remove glucose residues from the glycogen chain as glucose 1-phosphate. Further, when four glucose units remain before the branching point, the bifunctional debranching enzyme using the transferase activity will transfer the three glucose residues to an adjacent branch of the glycogen chain. After that, through the glucosidase activity, the debranching enzyme cleaves the α-1,6 linkage to release a free glucose from the branch point. Glycogen phosphorylase continues to the cleavage of glucose residues from the glycogen chain, producing glucose 1-phosphate.

The present review will address the role of glycogen metabolism in cancer and whether this metabolic pathway could be druggable and have therapeutic value combined with other anticancer therapies. Also, the reader is referred to earlier reviews on glycogen metabolism in more detail in normal and pathological conditions, for additional background.

## Glycogen biosynthesis: key proteins and enzymes

### Glycogenin

Glycogen biosynthesis requires the protein glycogenin, which is the core protein at the centre of glycogen granules. Glycogenin is an auto-glucosylating protein, which catalyses and initiates the formation of a short glucose polymer, leading to an α-1,4-linked chain of approximately 8–12 glucose residues [2, 3].

Elongation and branching of the glucose polymer by glycogen synthase and branching enzyme follow this reaction [4]. In humans, glycogenin exists in two isoforms, GYG1 (39 kDa, 350 aa) which is expressed predominately in skeletal muscle and GYG2 which is expressed in liver, heart and pancreas (55 kDa, 501 aa). Glycogenin contains a conserved domain of approximately 250 amino acids at its N-terminus, that is required for uridine diphosphate glucose binding and catalysis, and a highly conserved domain of 30–35 amino acids at its C-terminus which is required for glycogen synthase binding [5]. Glycogenin 1 deficiency causes a muscle glycogen storage disease, type XV [6]. Recently, Malfatti et al. described a new muscle glycogen storage disorder characterised by polyglucosan bodies that is due to either deficiency of glycogenin-1 or impaired interaction of glycogenin-1 with glycogen synthase [7]. Polyglucosan refers to abnormal amylopectin-like polysaccharides, which are less branched than normal glycogen and may aggregate into polyglucosan bodies [8]. Unlike normal glycogen, polyglucosan bodies are resistance to digestion with α-amylase [9]. Polyglucosan bodies have a fibrilar structure under electron microscopy and can be seen also in normal ageing in heart and central nervous system. Similar polyglucosan structures are also seen in deficiency of the glycogen branching enzyme deficiency mice [10]. Mutations in nine human genes, GYG1, GBE1, RBCK1, PFKM, EPM2A, EPM2B, PRDM8, PRKAG2 and GYS1, are known to be associated with polyglucosan structures [6]. It is clear that different forms of glycogen can be produced and that glycogenin is important for normal and functional glycogen particles. Whether glycogenin is important as therapeutic target in cancer is unknown.

### Glycogen synthase

**Fig. 2:**
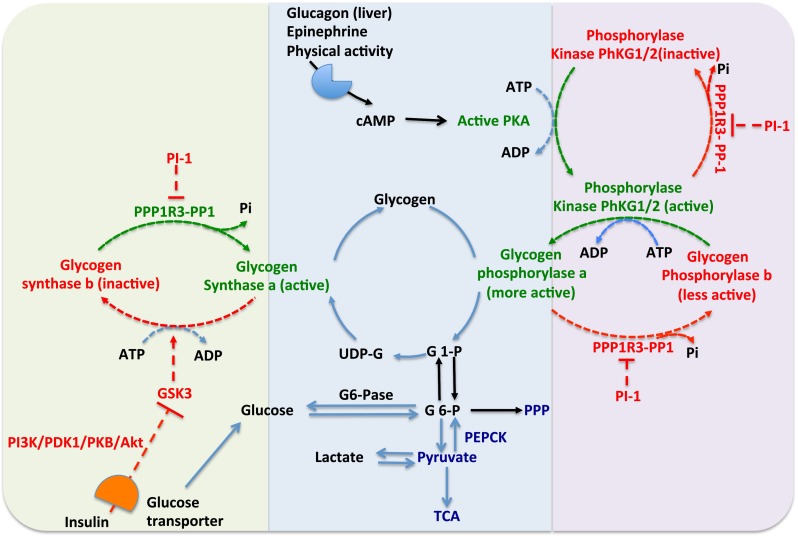
Regulation of glycogen metabolism by glycogen synthase and glycogen phosphorylase. Glucagon, epinephrine, physical activity induces cAMP via cognate receptors. cAMP activates protein kinase A (PKA). PKA converts the inactive phosphorylase kinase (PhK) to an active form. Also, PKA converts the active form of glycogen synthase a (GSa) to an inactive form of glycogen synthase b (GSb). The active PK phosphorylates and activates glycogen phosphorylase a (GPa). The protein phosphatase 1 (PP1) conjugated to the protein target to glycogen subunits (RRR1R3 family) dephosphorylates GPa and active PK and forms the less active GPb and inactive PK. GPa catalyses the degradation of glycogen to G1P and also inhibits the glycogen PP1 which converts the inactive glycogen synthase (GSb) to active glycogen synthase a (GSa). PP1 is regulated by another inhibitor called phosphoprotein phosphatase inhibitor (PI-1). PI-1 can be phosphorylated (activated) by PKA. PKA phosphorylation turns on GP and turns off GS. It also activates PI-1, which turns off the phosphatase (PP1) that would normally activate GS by dephosphorylating it. Insulin activates the insulin receptor tyrosine kinase, which further activates the PI3K and Akt and stimulates glycogen synthesis via inhibition of glycogen synthase 3 (GSK3), also activation of the PP1 and decrease of cAMP. Insulin stimulates the glucose uptake via translocation of glucose transporters to the plasma membrane. *Green text and lines* represent activation, whilst *red text and lines* represent inactivation

**Fig. 3:**
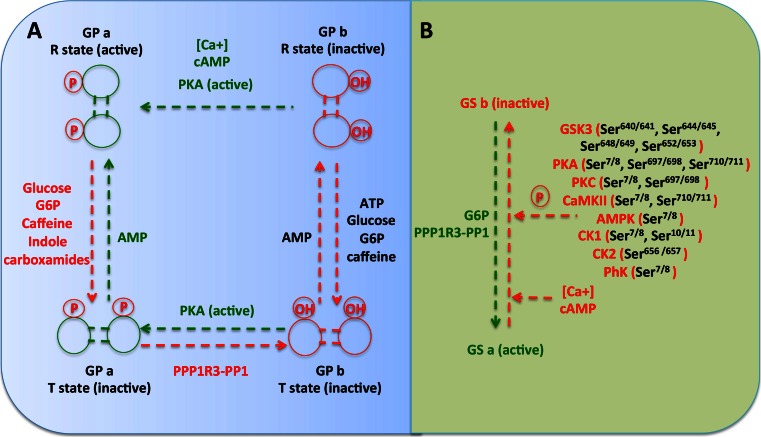
Regulation of glycogen phosphorylase and glycogen synthase. **a** Glycogen phosphorylase is controlled by phosphorylation and allosteric interactions. Glycogen phosphorylase convert to GPa by phosphorylation via phosphorylase kinase (PhK), whilst dephosphorylation via protein phosphatase 1 conjugated to protein glucogen target subunits (PPP1R3-PP1) turns back to GPb. GPa and GPb exist in equilibrium between a more active *R* state(*R*—relaxed) and less active/inactive *T* state (*T*—tense). Under allosteric control, AMP converts the inactive *T* states (GPa and GPb) to *R* states. Allosteric inhibitors such as ATP, glucose, glucose-6-phosphate, caffeine and indole carboxamides can alter the equilibrium back to the inactive *T* states of GP. **b** Glycogen synthase kinase 3 (GSK3), protein kinase A (PKA), protein kinase C (PKC), calmodulin-dependent protein kinase II (CaMKII), AMP-activated protein kinase (AMPK), casein kinase 1 (CK1), casein kinase 2 (CK2) and phosphorylase kinase (PhK) phosphorylate inactivate glycogen synthase via phosphorylation in different sites. Protein phosphatase 1 conjugated to protein target to glycogen subunits (PPP1R3-PP1) dephosphorylates the GSb and converts to active GSa form. Also, the ATP, glucose, glucose-6-phosphate and caffeine activate the GS, whilst calcium and cAMP deactivate the GS. *Green text and lines* represent activation, whilst *red text and lines* represent inactivation

In mammals, glycogen synthase exists in two isoforms, the glycogen synthase 1 (GYS1; 84 kDa, 737 aa), which is expressed in skeletal muscle and other tissues, and the GYS2 (81 kDa, 703 aa), which is expressed predominately in the liver. The liver isoform (GYS2, 81 kD) is about 70 % identical to the muscle isoform (GYS1, 84 kD) and has several phosphorylation sites near the N- and C-terminus [11]. GYS1 and GYS2 deficiency cause the muscle and liver glycogen storage disease type 0, respectively. Symptoms involved within those deficiencies are inability to form glycogen, muscle weakness, arrhythmia, sudden death and hypoglycaemia. Glycogen synthase exists in a phosphorylated (glycogen synthase b) and a dephosphorylated form (glycogen synthase a). Phosphorylation causes inactivation of the enzyme by decreasing the affinity for UDP-glucose [12, 13], whilst glucose-6-phosphate (G6P) is an allosteric activator of the phosphorylated form [14, 15] (Figs. 2 and 3). The crystal structure of the glycogen synthase in yeast was reported in 2010 in the presence and absence of the allosteric activator G6P [16]. Glycogen synthase exists as a tetrameric form and has direct interaction with glycogenin and glycogen branching enzyme in order to facilitate glycogen synthesis [5].

Glycogen synthase is regulated by multiple phosphorylation/dephosphorylation events and by allosteric effectors (Figs. 2 and 3). Further, these sites on glycogen synthase are phosphorylated in vitro by kinases such as protein kinase A, phosphorylase kinase, protein kinase C, protein kinases CK1 and CK2, glycogen synthase kinase 3 and AMP-activated protein kinase [12, 13].

Muscle glycogen synthase (GYS1) has nine well-characterised phosphorylation sites (rabbit/mouse 7/8, 10/11, 640/641, 644/645, 648/649, 652/653, 656/657, 697/698 and 710/711), whilst the liver isoform GYS2 has seven conserved phosphorylation sites and lacks the last two 697/698 and 710/711, which mainly regulated by protein kinase A in skeletal muscle [17, 18]. Phosphorylation and inactivation of the glycogen synthase occurred by various protein kinases (Fig. 3). Phosphorylation at Ser^7/8^ occurs by protein kinase A (PKA; also phosphorylates at Ser^697/698^ and Ser^710/711^), protein kinase C (PKC; also phosphorylates at Ser^697/698^), phosphorylase kinase (PhK), calmodulin-dependent kinase II (CaMK II; also phosphorylates at Ser^710/711^), mitogen-activated protein kinase (MAPK)-activated protein kinase 2 (MAPKPAK2) and AMP-activated protein kinase (AMPK) [19]. Hierarchical phosphorylation of Ser^7/8^ and Ser^10/11^ occurred also via casein kinase 1 (CK1).

Glycogen synthase kinase 3 (GSK3) acts predominantly in the phosphorylation of the glycogen synthase at different sites such as Ser^640/641^, Ser^644/645^, Ser^648/649^ and Ser^652/653^ [19]. Casein kinase 2 (CK2) phosphorylates glycogen synthase at Ser^656/657^ to initiate and create the recognition motif for the GSK3 to phosphorylate glycogen synthase at Ser^640/641^, Ser^644/645^, Ser^648/649^ and Ser^652/653^. Also, other protein kinases such as dual-specificity tyrosine phosphorylated and regulated protein kinase (DYRK) and Per-Arnt-Sim domain kinase (PASK) have been reported to directly phosphorylate GS at Ser^640/641^ [20, 21] and p38 MAPK Ser^644/645^ and Ser^648/649^ [22]. Mutational studies have confirmed that phosphorylation sites at Ser^7/8^, Ser^640/641^ and Ser^644/645^ are key regulators of the glycogen synthase activity [23, 24].

Muscle contraction stimulates GYS1 in order to rapidly restore glycogen content, which is used for energy supply through activation of glycogen phosphorylase. This occurs via dephosphorylation of GYS1 that is protein kinase B (PKB)/GSK3 independent [25] and dependent on the muscle specific protein phosphatase PP1G [26].

G6P allosterically activates glycogen synthase through the binding of the arginine-rich domain (Arg^579–591^). High concentrations of G6P can restore the activity of glycogen synthase, even if the enzyme is fully phosphorylated [27]. G6P regulates the activity of glycogen synthase not only by allosteric activation but also by making the protein a more suitable substrate for dephosphorylation by protein phosphatase 1 (PP1) [14]. Dephosphorylation of glycogen synthase is catalysed by PP1 bound to glycogen targeting subunits (PPP1R3), which remove a phosphate group by hydrolysis [28, 29]. Insulin activates the insulin receptor tyrosine kinase, which further activates the phosphatidylinositol 3-kinase (PI3K) and Akt pathways and stimulates glycogen synthesis via inhibition of glycogen synthase 3 (GSK3) and also by activation of the PP1 (Fig. 2) [30, 31]. Moreover, insulin stimulates glucose uptake via translocation of glucose transporters to the plasma membrane [30, 31]. Hypoxia and glucose starvation regulate the activity of glycogen synthase via the induction of the protein phosphatase 1, regulatory subunit 3 (PPP1R3C) [32, 33].

### Glycogen branching enzyme

The glycogen branching enzyme GBE1 (80 kDa, 702 aa) catalyses the transfer of α-1,4-linked glucose units from the outer ‘non-reducing’ end of a growing glycogen chain into an α-1,6 position of the same or neighbouring chain. Together, glycogen synthase and glycogen branching enzyme are both required for the globular and branched structure of glycogen, which is essential to increase its solubility by creating a hydrophilic surface and reduce the osmotic pressure within cells [34, 35]. The crystal structure and function of the human GBE1 have been described recently [36].

Glycogen branching enzyme deficiency is associated with an accumulation of insoluble polysaccharide particles, which lead to the autosomal recessive glycogen storage disorder type IV. Glycogen storage disorder type IV (GSDIV) is a severe disorder with variable onset age and clinical severity, including a classical hepatic form in neonates and children that progresses to cirrhosis (Andersen disease) [37], a neuromuscular form classified into four subtypes (perinatal, congenital, juvenile and adult onset) [38] and a late-onset adult polyglucosan body disease, a neurological disorder affecting mainly the Ashkenazi Jewish population [39]. The mechanism by which the glycogen branching enzyme orchestrates the glycogenin and glycogen synthase to form functional glycogen particles is not understood.

### Protein phosphatase 1 and glycogen targeting subunits

Protein phosphatase 1 (PP1; 35–38 kDa) is an essential eukaryotic protein serine/threonine phosphatase that regulates different cellular functions such as, cell division, glycogen metabolism, muscle contraction and protein synthesis [40, 41]. In mammals, there are three genes encoding the PP1 catalytic subunit, PP1α, PP1β/δ and PP1γ. The PP1γ gene encodes two proteins, PP1γ_1_ and PP1γ_2_, which arise through alternative splicing [40, 41].

Glycogen targeting subunits, PPP1R3 family proteins, have a major role in recruiting PP1 to glycogen and increasing the specific activity of PP1 towards specific glycogen enzymes such as glycogen synthase and glycogen phosphorylase [40–42]. One of the major functions of the PP1-PPP1R3 complex is the dephosphorylation and the activation of the glycogen synthase. There is a short conserved binding motif, the RVxF motif, in glycogen targeting subunits, which interact with the small hydrophobic groove on the surface of the protein phosphatase 1 [40–42].

At least seven genes encode the glycogen targeting subunits. The PPP1R3A G_M_/R_GL_ (124 kDa), gene that encodes the muscle specific subunit; the PPP1R3B G_L_ (33 kDa), gene that encodes the liver and muscle subunits; the protein target to glycogen (PTG) or Gc/R_5_/PPP1R3C (36 kDa), which is highly expressed in skeletal and cardiac muscle and liver; the G_D_/R_6_ PPP1R3D (33 kDa) and G_E_ PPP1R3E (31 kDa) which are most expressed in human skeletal muscle and heart [28]; the G_F_ PPP1R3F (79 kDa), which is expressed at high levels in the brain [43]; and the G_G_ PPP1R3G (38 kDa), which is expressed at high levels in the liver [44]. The N-terminal region of the glycogen targeting subunit is required for binding to PP1; the central region is required for binding to glycogen; and the C-terminal region is required for binding to glycogen phosphorylase, glycogen synthase and phosphorylase kinase.

Overexpression of protein target to glycogen subunit (PPP1R3C) in liver or fat cells significantly increases the glycogen levels [45, 46]. The three hepatic glycogen target subunits are much smaller compared to the muscle type PPP1R3A subunit, and this is due to their lack of a domain for interaction with the endoplasmic reticulum [29]. Three types of mechanism contribute to the glycogenic effects of the glycogen targeting subunits: (i) dephosphorylation of glycogen phosphorylase (inactivation) and glycogen synthase (activation), (ii) enhanced targeting of glycogen synthase and glycogen phosphorylase to the glycogen particle and (iii) stabilisation of glycogen synthase protein [47].

Recently, the glycogen target subunits have emerged as important components of glycogen metabolism. However, future research needs to highlight their importance in normal cell physiology and cancer and whether there is any therapeutic value for anticancer therapies. Why some tissues expressed more than one isoform and their effect on glycogen regulation under different metabolic stresses remain unclear. In hepatocytes, both PPP1R3B and PPP1R3C are induced in response to insulin, whilst PPP1R3G is repressed by insulin and induced by glucagon and glucocorticoids [44, 48]. Furthermore, the exact function of the glycogen targeting subunits as a sensor to orchestrate the interactions between the glycogen particles, glycogen enzymes and other enzymes such as glucokinase, glucose-6-phosphatase, is not well understood.

## Glycogen degradation pathways

**Fig. 4:**
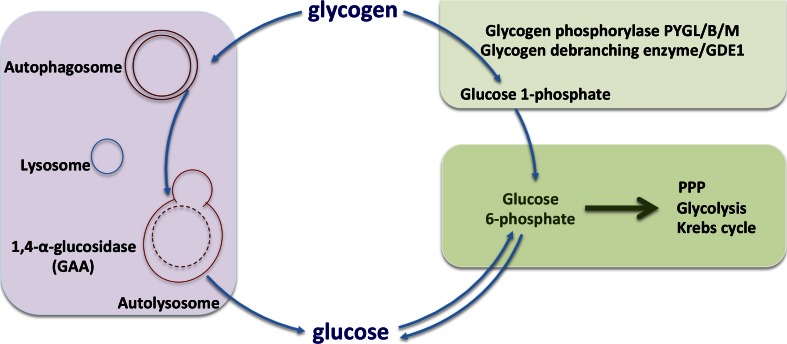
Glycogen degradation pathways. Glycogen can be degraded via two pathways. Glycogen phoshorylase and glycogen debranching enzyme produce glucose-1-phosphate (around 90 %) and free glucose (around 10 %) which converted to glucose-6-phosphate and enters glycolysis and TCA cycle. Autophagy-dependent glycogen degradation is very important in the newborns and produces free glucose via the lysosomal 1,4-α-glucosidase activity

Glycogen degradation can occur through two different pathways, the cytosolic and the autophagy-lysosomal pathway (Fig. 4). In the cytosol, glycogen degradation is mediated through two enzymes, glycogen phosphorylase and glycogen debranching enzyme. Whilst in the autophagy and lysosomal pathways, this process is mediated via the enzyme alpha acid glucosidase.

### Glycogen phosphorylase

Gycogen phosphorylase (GP) is the rate-limiting enzyme and in mammals comprises three isoforms, liver (PYGL, 97 kD), muscle (PYGM, 97 kD) and the brain (PYGB, 96.6 kD). For simplicity, hereafter glycogen phosphorylase will be referred to as GP for all isoforms. The liver enzyme serves the glycaemic demands of the body in general, whereas the brain and muscle isoenzymes supply glucose 1-phosphate just those tissues. Glycogen phosphorylase uses inorganic phosphate Pi to split glucose as glucose 1-phosphate from the polysaccharide chains of glycogen.

GP has an essential cofactor, pryridoxal phosphate (PLP), and is regulated by allosteric effectors and by phosphorylation of a single serine residue at the N-terminus [12, 13, 49–51]. Phosphorylation at serine-14 of glycogen phosphorylase b (GPb, less active) to glycogen phosphorylase a (GPa, active) is catalysed by phosphorylase kinase (PhK), which is activated by cAMP-dependent protein kinase A and increased levels of calcium [12, 13, 49–51]. GPs are dimers of two identical monomers, and allosteric effectors bind to five regulatory sites in each monomer, catalytic site (C site binds glucose, glucose 1-phosphate and inorganic phosphate), the purine nucleotide inhibitor or caffeine-binding site located near the active site, an AMP allosteric site that also bind ATP and G6P, glycogen site (G site) and the indole site that binds indole carboxamide ligands [12, 13, 49, 50]. PP1 in conjugation with the glycogen target subunit family proteins (PPP1R3 family) dephosphorylates the GP*a* to the inactive form, GP*b*. Both forms of GP can be found in the *T* (tense state) and *R* states (relaxed state), where *T* is the inactive state because it has a low affinity for substrate and *R* is the active state, given its greater affinity for substrate [12, 13, 49–51].

Phosphorylation and allosteric ligands (AMP, inorganic phosphate and glucose 1-phosphate) stabilise the *R* state, whilst glucose, ATP, purine nucleosides and G6P stabilise the less active *T* state (Fig. 3). Moreover, glucose and other ligands that stabilise the *T* state promote the dephosphorylation of the GPa to GPb by PP1. The phosphorylation control of GP is a response to messages from the extracellular environment, signalled by hormones, whilst allosteric control is a response to intracellular sensors of the cell metabolic status [12, 13, 49, 50]. During physical activity, hormonal stimulation (glucagon and epinephrine) activates the enzyme phosphorylase kinase, which in turn phosphorylates and converts the less active form GPb into the more active form, GPa (Fig. 2) [12, 13, 49, 50]. A recent discovery is that glycogen phosphorylase is negatively regulated via acetylation. Acetylation of lysine 470 and 796 inhibited glycogen phosphorylase activity and enhanced its interaction with PPP1R3C-PP1, thereby promoting glycogen phosphorylase dephosphorylation [52]. Acetylation of glycogen phosphorylase was low in the fasted state, and it was increased by high glucose and insulin, whilst exposure of cells to glucagon had the converse effect and promoted deacetylation [52].

Deficiency in muscle glycogen phosphorylase PYGM causes a glycogen storage disease type V (also known as GSDV or McArdle disease). People with GSDV typically experience muscle fatigue and pain and sometimes severe symptoms such as rhabdomyolysis during prolonged or intense exercise. Deficiency in liver glycogen phosphorylase PYGL causes inability to break down liver glycogen and a glycogen storage disease type VI (also known as GSDVI or Hers disease). People with GSDVI have symptoms such as hepatomegaly, growth retardation, ketotic hypoglycaemia and post-prandial lactic acidosis.

### Glycogen debranching enzyme

The glycogen debranching enzyme amylo-α-1,6-glucosidase, 4-α-glucanotransferase (AGL) (174.76 kDa, 1532 aa) binds to glycogen through its C-terminal domain and possesses two different independent catalytic activities, which occur at different sites on the protein [53–55].

Glycogen phosphorylase is responsible for the cleavage of the α-1,4 glycocidic bonds and release G1P monomers that can further metabolise. When glycogen phosphorylase digests a glycogen branch down to four glucose residues, it will not be able to cleave residues further along or break α-1,6 glycocidic bonds at glycogen branch points.

Glycogen debranching enzyme assists the further cleavage of the glucose residues. First, via the 4-α-D-glucanotransferase or glucosyltransferase activity transfers/relocates the three glucose residues from the four-residue glycogen branch to a nearby branch. This leaves one glucose unit joined to the glucose chain branch point through an α-1,6-glycosidic linkage [53–55]. Second, via the amylo-α-1,6-glucosidase activity cleaves the remaining alpha-1,6 linkage and produces free glucose and a linear of glycogen [53–55].

Deficiency in glycogen debranching enzyme results in glycogen storage disease type III (Cori’s disease) with an accumulation of abnormal glycogen with short outer chains. It is clinically characterised by hepatomegaly, hypoglycaemia, short stature and variable myopathy. Glycogen storage disease type III includes different forms. GSD type 3A patients lack glycogen debranching enzyme activity in both liver and muscle, whilst GSD type 3B patients are enzyme deficient in liver only. In rare cases, selective loss of only one of the two debranching activities, glucosidase or transferase, results in GSD type 3C or type 3D, respectively [56].

### Autophagy and lysosomal pathways

Autophagy is a fundamental intracellular process for the degradation and recycling of cytoplasmic constituents through lysosomes. The recycled materials are major inputs for multiple anabolic and biosynthetic pathways in cells. Glycogen autophagy (glycophagy) is the autophagic sequestration and degradation of glycogen to support glucose homeostasis [57, 58]. Glycogen autophagy is a very important process for the production of glucose of newborn animals [57, 59–61]. In newborns, glycogen autophagy is induced by glucagon during post-natal hypoglycaemia and is inhibited by insulin and parenteral glucose [57].

An accumulation of autophagic and lysosomal vesicles containing glycogen has been observed in several myopathies such as Pompe disease, Danon disease, infantile autophagic vacuolar myopathy and drug-induced vacuolar myopathies caused by treatment with chloroquine or hydroxychloroquine [62, 63]. However, it remains unclear how glycogen metabolism connects to the pathology of the diseases. Mice lacking the lysosomal enzyme acid α-glucosidase GAA^−/−^ in skeletal muscle, a model for Pombe disease, have shown massive accumulation of glycogen in lysosomes, autophagosomes and late endosomes which are major characteristics of the diseases [64]. In the same model, loss of Atg5 diminished the glycogen built up in autophagosomes but make the clinical phenotype worse [65]. Further, loss of the autophagy protein Atg7 decreased glycogen by 50–60 %, whilst enzyme replacement therapy of GAA normalised the glycogen levels in the autophagy deficient mice, an outcome not observed in Pompe mice with genetically intact autophagy [66].

Another study found that CQ-induced myopathy can be improved by decrease of autophagy or glycogen synthesis, the latter possibly due to a direct role of glycogen synthase in regulating autophagy through its interaction with the Atg8 family [67]. More recently, a study by Farah et al. [68] found that pharmacological induction of autophagy corrects hepatic glycogen and lipid accumulation in glucose-6-phosphatase deficiency mice G6Pase^−/−^, an animal model of von Gierke’s disease or GSDIa, suggesting the importance of autophagy in glycogen and lipid metabolism as a new therapeutic strategy in GSDIa.

The exact mechanism by which glycogen sequestered by autophagy and transferred to lysosomes and whether any defects on that process lead to diseases remain unknown. GYS1 has been identified as potential interactor of GABARAPL1, using mass spectroscopy [69]. More recently, Zirin et al. [67] identified three putative LIR motifs conserved between *Drosophila melanogaster* glycogen synthase and its mammalian orthologs, VAHFHE (residues 187–192), EFQNL (residues 303–307) and DWRTL (residues 608–612), which interact with Atg8 under starvation conditions. In this respect, the metabolic interaction and key protein players between autophagy and glycogen particles need to be further investigated.

### Liver

The glycogen content in liver is around 100 g in 1.5-kg tissue [70]. The liver maintains normal blood glucose levels by rapid clearance of glucose from the portal vein in the absorptive state after a meal and by controlling production of glucose in the post-absorptive state at a sufficient rate to maintain euglycaemia [12]. The liver accumulates glycogen in large amounts and releases it slowly to maintain blood sugar levels. Glycogen synthesis is performed in the cytosol from extracellular glucose transported into the cells through glucose transporters (direct pathway) or from gluconeogenic substrates, such as lactate and amino acids (indirect pathway). The indirect pathway occurs mainly in the liver, following either the intrahepatic or extrahepatic conversion of gluconeogenic precursors into glucose [71].

### Muscle

In humans, 80 % of glycogen stored in skeletal muscle just because skeletal muscle accounts for 40–50 % of body weight, whilst the concentration of the glycogen is around 80–150 mmol/kg. Each glycogen granule contains approximately 30,000 glucose residues with numerous non-reducing ends for rapid access to glucose [72]. In skeletal muscle, up to 90 % of glucose load is converted to glycogen and the amount of glycogen stored is higher in type II fibres (fast switch) compared with the type I (slow twitch). In skeletal muscle, distinct pools of glycogen with distinct characteristics and functions in muscle contractility have been identified [73, 74]. Electron microscopy studies have shown that glycogen pools localised in the subsarcolemmal, in the intermyofibrillar glycogen located between the myofibrils close to sarcoplasmic reticulum and mitochondria and in the intramyofibrillar glycogen [73, 74]. It is well known that glycogen correlates with ability of skeletal muscle capacity to perform prolong and high-intensity exercise [75]. Skeletal muscles are unable to produce and release glucose, because they lack the glucose-6-phosphatase enzyme; thus, muscle glycogen is mainly local energy deposits for muscle contractions such as exercise. However, skeletal muscles are able to produce high levels of lactate, up to 30 mM, through the glycogen shunt and which can be transported to other tissues such liver and brain as an energy source.

### Brain

The average glycogen content in brain is around 3–12 μmol, which is much less compared to liver and muscle [76, 77]. Glycogen is very important in brain function such as synaptic activity, memory formation, sensory stimulation and sleep and wake cycles [77–79]. Also, glycogen is very important in protecting the brain function under stress conditions such as hypoglycaemia [80], hypoxia/ischaemia, exhaustive exercise [81] and seizures [82]. Further, increased glycogen stores in astrocytes are able to preserve/protect neuronal function and viability under hypoglycaemia. It has been proposed that astrocytes are able to provide energy to neurons via a mechanism called the glycogen shunt where lactate is produced in astrocytes and then is used by neurons for their energy requirements.

Although it was thought that glycogen in astrocytes through the glycogen shunt supports the activity of neurons, it is well established that neurons have an active glycogen metabolism, which contributes to tolerance to hypoxia [83]. Neurons synthesise and degrade small amounts of this polysaccharide continuously. They do not use it as an energy store but as a rapid and small, but constant, source of energy. Glycogen metabolism is able to generate 3ATP, whilst 2ATP is generated from the free glucose. Also, neurons through transmitters and neuromodulators stimulate the mobilisation of the astrocyte glycogen stores, which are converted to lactate to be taken up and utilised by neurons [84, 85]. Glycogen accumulation in neurons during aging is an evolutionarily conserved process from flies to mammals, and Laforin disease resulting from rare mutations in malin and laforin increases the rate of this process [86]. Laforin is a phosphatase of glycogen synthase that can directly bind glycogen through a carbohydrate-binding domain as well as other glycogen metabolising proteins [17]. Malin is an E3-ubiquitin ligase that is recruited to its substrates through the interaction with Laforin [17]. Furthermore, Laforin and malin form a complex, and with the glycogenolytic enzymes, glycogen debranching enzyme 1 (AGL1) and brain isoform glycogen phosphorylase orchestrate the degradation of the polyglucosan bodies [87]. Brain glycogen or glycogen-like inclusions accumulate in several pathologies such as Pombe disease [88], Lafora disease [89], Alzheimer disease [90], amyotrophic lateral sclerosis [91] and adult polyglucosan body disease [9].

The mutations in genes involved in glycogen metabolism lead to glycogen storage diseases, which affect primarily the liver, skeletal muscle, heart and the central nervous system and kidneys. Glycogen storage diseases are classified according to their individual enzyme deficiency, each of which enzyme regulates synthesis or degradation of glycogen.

These data indicate that the complexity of glycogen metabolism at a tissue level and key issues are whether tumours and their metastases reflect their tissue of origin or destination or adopt different components for maximum survival in stress conditions.

## Methods for assessing glycogen stores and turnover

Several techniques have been described for detecting and assessing the level of glycogen in cells, tissues and entire organs, such as electron microscopy, immunohistochemical, biochemical, live fluorescence imaging and radioisotope methods.

Electron microscopy is the only technique able to show in detail the fine structure of the cell and its various compartments. The fine structure of glycogen was first described by Revel and colleagues in 1960, as roughly circular granules from 150 to 400 A in diameter [92]. Today, electron microscopy is a powerful tool to analyse glycogen within the cell and its localisation. However, due to the lack of expertise, cost-effectiveness and time effectiveness, this technique cannot be used routinely. Routinely, the presence of glycogen can be examined histochemically in both cryosections and formalin-fixed, paraffin-embedded sections by the periodic acid-Schiff (PAS) reaction [93]. Glycogen subcellular distribution can be studied by immunohistochemistry and immunofluorescent techniques by using a specific antibody against glycogen [94–96], which was developed by Dr. Baba [97]. Notably, it is very important to maintain optimal preservation of glycogen in biopsies. Up to 70 % of glycogen can be lost using formalin fixation, and this is due to the soluble nature of the predominant form of glycogen in the cytoplasm [98].

Three biochemical methods have been used for analysis of glycogen content in tissue homogenates: (i) acid hydrolysis of the tissue followed by enzymatic analysis of glucose, (ii) enzymatic hydrolysis with amylo-α-1,4-α-1,6-glucosidase followed by analysis of glucose and (iii) analysis of glucose-1-P produced by degradation of glycogen with phosphorylase and debranching complex [99].

The 2-NBDG is a fluorescent glucosamine derivative that enters the cells through glucose transporters, is phosphorylated by the enzyme hexokinase and cannot be metabolised through glycolysis but is incorporated into glycogen granules. Therefore, this probe is suitable for glycogen quantification and imaging [100]. However, this technique is ideal only for in vitro measurements and cannot be used for glycogen in vivo determinations, due to the limited tissue penetration of the emitted green fluorescence. More recently, a new technique has been developed to detect de novo glycogenesis in tumour cells in culture and in tumour xenografts in vivo, by using a novel radiotracer, (18)F-N-(methyl-(2-fluoroethyl)-1H-triazole-4-yl)glucosamine (18F-NFTG) [101]. This technique shows great promise for future clinical applications, allowing tumour stratification according to glycogen content and correlation with treatment response.

## Glycogen metabolism in cancer progression and microenvironment

Cancer metabolism and the metabolic reprogramming during the adaptive process of cells within tumour microenvironment and resistance to anticancer therapies have been well recognised in the last decade [102]. Genetic factors such as oncogenes (i.e. MYC) and tumour supressors and microenvironmental factors such as hypoxia and acidosis could regulate glycogen metabolism in cancer cells. Further, cancer-associated fibroblasts are a major component of the tumour microenvironment and have a major role in cancer progression by providing cancer cell metabolic intermediates such as lactate, amino acids, ketone bodies and various signalling molecules [103, 104]. Glycogen metabolism is so far uninvestigated in this role. Other stromal cells include immune infiltrates, which have been of great of interest in cancer progression, invasion and therapy. Apart from early studies on differential glycogen expression in T and B cells, this remains to be investigated [105]. Taken together, we hypothesise that glycogen metabolism is a major energy source within the tumour microenvironment as well as in the tumour and its response to chemotherapy and radiation therapy.

Large quantities of glycogen have been described in various cancer cell lines such as breast, kidney, uterus, bladder, ovary, skin and brain cancer cell lines and in particular cells undergoing neoplastic transformation [106–109]. Glycogen metabolism was crucial for maintenance in vitro of hepatomas [110]. Further, a ‘clear cell carcinoma’ represents a small number of cases in different cancer types, i.e. breast, which are characterised by high quantities of glycogen, and aggressive clinical behaviour [111]. Further, to our knowledge, there is no information available on the prevalence of cancer in glycogen storage disease patients. The only data available regards to occurrence of liver adenomas (with occasional development of carcinomas) in some of the disorders. However, the pathogenesis of these lesions is unclear, and in some instances, it has been linked to the simultaneous occurrence of metabolic derangements [112].

Hypoxia is a hallmark of the tumour microenvironment and is characterised by low oxygen concentration as a result of abnormal blood vessels, defective blood perfusion and limited nutrients. Thus, under hypoxia, cancer cells must adapt in order to maintain tumour growth. It is well established that glycogen has a crucial role to promote cell survival under hypoxic conditions in normal and cancer cells [113–117]. Pescador et al. [116] showed that hypoxia increases glycogen accumulation in myotubes, hepatocytes and hepatoma cells, through hypoxia-inducible factor 1α (HIF1α) stabilisation. Importantly, the investigators found that GYS1, UTP:glucose-1-phosphate uridylyltransferase (UGP2) and 1,4-alpha glucan branching enzyme (GBE1) were significantly increased after hypoxia conditions. Further, by knocking down either HIF1α or GYS1 attenuated hypoxia-induced glycogen accumulation, whilst GYS1 overexpression was sufficient to mimic this effect, suggesting that GYS1 regulation by HIF1α plays a central role in the hypoxic accumulation of glycogen [116]. Also, hypoxia preconditioning protects cells during anoxia whilst knockdown of GYS1 expression impairs hypoxic preconditioning and protection, suggesting the important role of glycogen metabolism in acute and chronic hypoxia [116].

Similarly, the study by Shen et al. [32] found that hypoxia induced accumulation of glycogen 24 and 48 h after exposure. Further, they found that PPP1R3C induction correlates with a significant glycogen accumulation in MCF7 cells under hypoxia whilst knockdown of either HIF1α or PPP1R3C attenuated hypoxia-induced glycogen accumulation significantly. Pelletier et al. [115] established that hypoxia-induced accumulation of glycogen stores are rapidly mobilised in cells that are starved from glucose, whilst normoxic control cells exhibit a high rate of cell death following glucose removal. They found that phosphoglucomutase 1 (PGM1), which converts the glucose-6-phosphate to glucose-1-phosphate, was upregulated in hypoxia conditions via HIF1α. Hypoxia induced and enhanced accumulation of glycogen in PIK3CA mutant in human ovarian clear cell carcinoma [117]. In particular, the glycogen synthase 1, dephosphorylation of GYS1, protein target to glycogen subunit and glycogen content were increased 24 and 48 h after hypoxia conditions [117]. In another study, we found that GYS1, PYGL, GAA, GBE, PPP1R3B and PPP1R3C were upregulated in U87MG cell lines under hypoxia conditions [113].

Glycogen metabolism may have an important role for cancer cell survival under nutrient starvation conditions. Protein target to glycogen subunit PPP1R3C (PTG) protects liver cancer cells in the absence of glucose via regulation of oxidative stress and autophagy, whereas silencing PPP1R3C further promotes cytotoxicity [33]. Glycogen phosphorylase brain form (PYGB) was found to be important also in nutrient starvation conditions in cancer cell survival. In pancreatic 2-deoxy glucose resistance cell lines, PYGB was low suggesting a reduced rate of glycogen turnover, whilst knockdown of PYGB in parental cell lines resulted in cell death under 2-DG treatment [118]. Another study with gastric cancer cell lines found that PYGB activity and glycogen breakdown were increased upon serum starvation conditions, resulting in reduced apoptosis and cancer cell survival [119].

Downregulation of glucose-6-phosphatase in glioblastoma cell lines decreased cell viability, invasion and migration as well as decreased cell survival under 2-deoxy-glucose treatment [120]. This resulted in accumulation of glucose-6-phosphate, increased activity of GYS1, downregulation of PYGL and increased glycogen accumulation [120].

Cancer genomic data has shown that the glycogenic enzymes GYS1, GYS2 and GBE1 are upregulated in 18 % in AML patients and with significantly poor survival outcome [121]. Knockdown of GYS1 increased activation of AMPK and decreased growth of leukaemia cells in vitro and in vivo. Cheng et al. [122] found that Rab25, a small GTPase involved in endosomal recycling, enhance survival during nutrient stress by preventing apoptosis via increased Akt activation and subsequent glucose uptake, glycogen accumulation and improved cellular bioenergetics. Also, the PhKG1 (the γ-subunit) of the holoenzyme phosphorylase kinase, involved in activation of glycogen phosphorylase, has been shown to be upregulated in several human tumour samples, also involved in tumour progression, angiogenesis and tumour metabolism [123]. This study indicates that PhKG1 could be a potential target candidate therapy.

Recently, the glycogen debranching enzyme AGL was shown to have tumour suppressor function in bladder cancer, and that loss of AGL leads to increased tumour growth in xenograft models of bladder cancer [124, 125]. Loss of AGL leads to decrease in normal cellular glycogen with an increase in abnormal glycogen structures (e.g. limit dextrin) and increase in glucose consumption and lactate production [124]. Lowering AGL enhanced tumour growth by increasing glycine synthesis through increased expression of serine hydroxymethyltransferase 2 (SHMT2), whilst depletion of the SHMT2 rescues the effects of tumour growth of bladder cancer cells without AGL [124]. It is interesting to note here that the effect of the AGL in tumour growth was independent of its enzymatic activity, suggesting that AGL has an unknown non-enzymatic function. Another study found that hyaluronic acid 2 (HAS2) is the driver of tumour growth of bladder cancer with low AGL, suggesting a preclinical rational for personalised targeting in patients with low AGL expressing bladder tumours [126]. Recently, Chen et al. found that metastasis of breast cancer cells to brain exhibit metabolic reprogramming with a glycogenic future and ability to store glycogen [127]. In the same study, brain metastases from human breast cancer patients expressed higher levels of fructose-1,6-biphosphate and glycogen compared to the breast primary tumours [127]. This metabolic switch enables cancer cells to survive under adverse conditions such as glucose deprivation, suggesting that glycogen metabolism might be a novel target for brain metastasis from breast cancer.

The accumulation of glycogen granules in renal cancer cells is a well-known feature of the ‘clear cell’ versus ‘papillary’ phenotypes but has not been developed as a specific therapy target [128, 129]. HIF-dependent regulation of glycogen synthesis has also been confirmed in the human renal clear cell carcinoma cell lines RCC4 and 786-O, which are characterised by constitutive HIF1α and/or HIF2α activation, due to a defect in the tumour suppressor protein and HIF-negative regulator von Hippel-Lindau (VHL) [130]. Similarly, it should be investigated whether altered glycogen metabolism plays any role on the development of renal clear cell carcinomas driven by VHL mutations.

Taken together, all the above studies show that cancer cells are able to accumulate glycogen as a deposit source of energy to enable survival under adverse conditions such as hypoxia and glucose deprivation as well as sustain metastases.

## Pharmacological modulators of glycogen metabolism

The evidence that glycogen metabolism is reprogrammed in cancer cells indicates that targeting of glycogen metabolism could represent a new strategy for cancer treatment. Preclinical and clinical studies will be required to identify the best candidates to target, and the clinical features associated with glycogen storage disorders can assist in predicting the toxicity associated with depletion of the different glycogen metabolism enzymes. In particular, GP defects in Hers’ disease patients are associated with hepatomegaly, growth retardation, ketotic hypoglycaemia and post-prandial lactic acidosis. However, despite those, toxicities might have a therapeutic window and limit toxicities by targeting the liver glycogen phosphorylase. In Table 1, the pharmacological modulators and specific inhibitors of glycogen metabolism are shown.

**Table 1:** Pharmacological modulators and specific inhibitors of glycogen metabolism

Glycogen phosphorylase inhibitors	Pharmacological modulators of glycogen metabolism	GAA/1,4-α-glucosidase inhibitors
(i) Active site inhibitors	Sodium tungstate (increases glycogen synthesis)	Acarbose
e.g. DAB	Metformin (depletes glycogen)	Miglitol
(ii) AMP site inhibitors	Lithium (stimulates or inhibits glycogen synthesis)	Voglibose
e.g. BAY1807	Valproate (decreases glycogen content)	
BAYR3401	Dichloroacetate (increases glycogen accumulation)	
(iii) Indole carboxamide site inhibitors		
e.g. CP91149		
Ingliforib (CP368296)		
CP316819		
CP320626		
(iv) Purine nucleoside site inhibitors		
e.g. Olefin derivatives		

Four major classes of glycogen phosphorylases inhibitors. Commercially available inhibitors that block the activity of the GAA enzyme. Compounds which have shown in the literature that modulates glycogen metabolism

Lithium (LiCl) is commonly used for the treatment of mood and bipolar disorder and has recently been evaluated as an anticancer drug. Lithium treatment can inhibit glycogen synthesis and decrease glycogen content in cortical cultured astrocytes [131] and in the rat liver [132]. On the other hand, lithium treatment stimulates glycogen synthesis/accumulation and glycogen synthase activity in the rat diaphragm [133] and in salivary glands of the rat [134]. Lithium is under evaluation in clinical trial of prostate cancer (clinicaltrials.gov; NCT02198859). Valproate, a histone deacetylase inhibitor, increased the glycogen phosphorylase brain isoform and decreased glycogen accumulation in the skeletal muscle of McArdle disease [135]. Valproate is under evaluation in clinical trials of advance cancers in combination with temsirolimus and bevacizumab (clinicaltrials.gov; NCT01552434). Also, an antidiabetic and antiobesity agent, sodium tungstate, increased glycogen synthesis and accumulation in hepatocytes [136, 137]. Further, cardiac steroids (i.e. bufalin) induced the formation of glycogen-microtubule structures via a complex process that requires different signalling pathways [138].

Metformin, an old antidiabetic drug, has been widely used in cancer research as a potent combinational anticancer therapy. However, the mechanism of action in cancer cells is not very well understood yet. Recently, it was found that metformin depletes glycogen content in myeloid leukaemia cells, suggesting that metformin might block the synthesis of glycogen and activates the degradation pathways [121]. Another study found that ionising radiation resulted in accumulation of glycogen in lung, prostate and breast cancer cell lines, whilst metformin rescued the accumulation of glycogen and sensitised the cells to ionising radiation [139]. Metformin is now been used in various clinical trials in combination with anticancer therapy. Dichloroacetic acid (DCA) increases hepatocellular glycogen accumulation through a PI3K-dependent mechanism that does not involve PKB/Akt and is, at least in part, different from the classical insulin-stimulated glycogenesis pathway [140].

Furthermore, specific inhibitors for the rate-limiting enzymes for glycogen metabolism would be the best approach to target this pathway. However, only the glycogen phosphorylase enzyme has been studied as a potential therapeutic target for type 2 diabetes. GP is the key enzyme that drives glycogenolysis and therefore comprises an important target in the attempt to reduce post-prandial glucose levels by lowering hepatic glucose production. Extensive reviews on a range of GP inhibitors and proposed sites of actions have been reported [12, 13, 49, 141–145]. Binding sites for physiological and pharmacological ligands have been identified by crystallography. The following four major regulation sites have been used to develop glycogen phosphorylase inhibitors: the catalytic-active site (C site), nucleotide-binding site (adenosine monophosphate (AMP) site), purine nucleoside site (P site) and the indole site (Table 1) [49]. The presence and absence of an allosteric ligand such as glucose and AMP can influence the inhibitory activity; thus, researchers need to be careful when using the glycogen phosphorylase inhibitors.

### Active site inhibitors

Glucose analogues such as N-acetyl-β-D-glucosamine and the bicyclic compound glucopyranose spirohydantoin have been synthesised; however, these two analogues have lower affinity for liver than muscle isoforms of GP. The most potent active site inhibitor of liver glycogen phosphorylase a is azasugar 1,4-dideoxy-1,4-amino-D-arabinitol (DAB) [146, 147]. DAB is a potent inhibitor of GPa in hepatocytes in both in the absence and presence of glucagon, with half-maximal effect at 1–2 μM and greater than 80 % inhibition of glycogenolysis at 5–20 μM [15]. DAB is also an indirect inhibitor of glycogen synthesis [148].

### AMP site inhibitors

Ligands of the AMP activator site cause both inactivation and allosteric inhibition of glycogen phosphorylase a. A pro-drug, the dihydropyridine derivative (BAY3401), is metabolised to 1,4-dihydropyridine-2,3-dicarboxylate (BAY1807) and binds to the AMP site and causes the conversion of GPa to GPb in liver and skeletal muscle [149]. Researchers at Novo-Nordisk [150], Merck [151] and Sanofi Aventis [152, 153] have identified several other compounds that bound to the AMP site, with EC50 values in the nanomolar range. Phthalic acid derivatives bind at the AMP active site and are potent inhibitors of liver and muscle GPa [150]. Diacid analogues that bind at the AMP site not only are very potent but also have approximately tenfold selectivity against liver versus muscle GP in in vitro assays [151]. Acyl urea derivatives, which bind at the AMP active site, were discovered as a novel class of inhibitors for glycogen phosphorylase [153].

### Indole carboxamide site inhibitors

There is one indole site to each subunit, and in the native *T* state of GPb, the site is occupied by 30 water molecules [13]. This site is referred to as the indole site since almost all inhibitors that bind to the site have been based on an indole-2-carboxamide scaffold. Researchers from Pfizer [154–158], Merck [159] and AstraZenca [160–162] have identified several indole carboxamide site inhibitors. One of these inhibitors is ingliforib (CP368, 296), which inhibits the GP isoforms with IC50 values of 52 nM (liver), 150 nM (brain) and 352 nM (muscle).

### Purine nucleoside site inhibitors

The purine nucleoside site inhibitors include purines (e.g. caffeine), flavopiridol, nucleosides (e.g. adenosine) and nucleotides (e.g. AMP, IMP, ATP, NADH and FAD) [12, 13, 49, 50]. Flavopiridol, an antitumour drug and inhibitor of cyclin-dependent kinases, was identified as a high-affinity ligand of the purine nucleoside site [163, 164]. Further olefin derivatives of flavopiridol have been synthesised with similar potency on glycogen phosphorylase but high potency in hepatocytes [165].

## Targeting components of glycogen metabolism in cancer and clinical implications

**Fig. 5:**
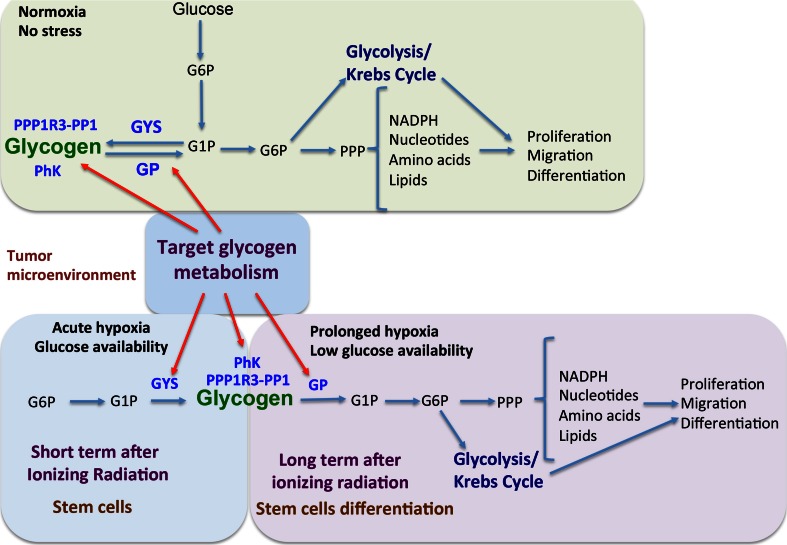
Targeting glycogen metabolism as a strategy in cancer therapy. Targeting glycogen synthase, glycogen phosphorylase, phosphorylase kinase and glycogen target subunits as well as other players in glycogen metabolism could enhance anticancer therapy

In Fig. 5, a schematic presentation of targeting glycogen metabolism as a combinational strategy for anticancer therapy is shown. Glycogen metabolism is upregulated after bevacizumab treatment in glioblastoma U87MG xenograft model [113]. In particular, the gene expressions of GYS1, PPP1R3B, PPP1R3C, GBE, PYGL and GAA, together with the hypoxia marker CA9, found to be significantly upregulated in U87MG xenografts treated with bevacizumab [113]. Bevacizumab treatment as an angiogenic therapy would increase hypoxia within the tumour and eventually an adaptive response of cancer cell, e.g. accumulation of glycogen as an energy deposit. Furthermore, from the in vitro experiments, the observation that glycogen accumulates under hypoxia conditions as a mechanism of cancer cell survival suggests the idea that combinational therapy of targeting glycogen metabolism together with bevacizumab treatment could provide a potent strategy for anticancer therapies. Targeting PYGL for cancer therapy would likely have a therapeutic window and limited side effects.

Similar targeting of the GYS1 could be another potential anticancer therapeutic approach for limited side effects, since mice lacking the skeletal muscle GYS1 are functional and physically active. An antisense approach to target glycogen synthase 1 in C6 rat glioma cell lines has been described by Ardourel et al. [166], with great therapeutic value.

Bladder cancer cells with low AGL expression were found to be more sensitive to glucose deprivation and glycolysis inhibition by 2-deoxygl-D-glucose [124]. Similarly, another study found that human pancreatic cancer cell lines with low levels of glycogen phosphorylase B (PYGB) are more sensitive to glucose deprivation conditions, suggesting that PYGB could be a therapeutic target in those cancers with limited glucose levels [118]. Moreover, autophagy might sustain the glycogen breakdown; thus, by blocking both pathways could provide synergism as anticancer treatment.

Glycogen breakdown supports the pentose phosphate pathway, which generates nucleotides required for proliferation and DNA repair, as well as NADPH, which is an important reducing agent for reactive oxygen species (ROS) scavenging and nucleotide, amino acid and lipid synthesis. Considering this major role of glycogen metabolism in the pentose phosphate pathway, combination treatment of glycogen inhibition with DNA damaging agents, ROS generating agents, mitochondria inhibition agents and ionising radiation might be a potent strategy for anticancer treatment. Also, most of the above molecules involved in glycogen metabolism have not been tested in cancer cell lines to assess activity or differential effects based on driver mutations of metabolism.

## Conclusions

In conclusion, therefore, glycogen metabolism has emerged as a complex pathway intersecting with many key metabolic pathways including the Krebs cycle, pentose shunt, glycolysis and lipid biosynthesis. It represents a major store of high-energy glucose which does not require ATP for activation. Thus, under stressful conditions, it may be particularly important for survival of tumour cells. Since this is regulated in hypoxia and by so many different kinase pathways, the final affects will be complex. However, there exists a wealth of drugs and kinase inhibitors that could be exploited to target this pathway and it is important to use the most appropriate screening conditions for their effect. It is most likely that those representing the microenvironment in vivo, such as low glucose and hypoxia and acidosis, would be key. It would be important to test these in in vivo models under such stressful conditions and to try and repurpose drugs that never went into the disease area for which they were planned. This still represents a major problem for interactions between academia and the pharmaceutical industry, to have access to these molecules with appropriate possibilities for licensing and development.
